# Discovery of Novel Doxorubicin Metabolites in MCF7 Doxorubicin-Resistant Cells

**DOI:** 10.3389/fphar.2019.01434

**Published:** 2019-12-06

**Authors:** Xu Wang, Renjie Hui, Yun Chen, Wentao Wang, Yujiao Chen, Xiaohai Gong, Jian Jin

**Affiliations:** School of Pharmaceutical Sciences, Jiangnan University, Wuxi, China

**Keywords:** drug resistant, doxorubicin, intracellular drug metabolism, LC-MS/MS, breast cancer

## Abstract

Doxorubicin (DOX) is metabolized to a variety of metabolites *in vivo*, which has been shown to be associated with cardiotoxicity. We speculate that metabolic processes are also present in tumor cells. A LC-MS/MS method was developed to detect intracellular metabolites. Drug resistant tumor cells with high drug stress tolerance and metabolically active are suitable as materials for this study. Our results show difference in drug metabolites between the wild-type and drug-resistant cells. Three novel doxorubicin metabolites were discovered after the LC-MS/MS analysis. All these metabolites and their profiles of metabolites are totally different from that in liver or kidney *in vivo*. Our results suggest that tumor cells and drug-resistant tumor cells have a unique drug metabolism pathway for doxorubicin.

## Introduction

Some chemotherapeutic drugs need to enter the target tumor cells to produce cytotoxic effects. The forms of intracellular drugs are important for the pharmacological effects and the occurrence of drug resistance. In our previous studies, it was found that intracellular drugs bind to various proteins and affect their function (Chen et al., 2017). Enzymes that bind to drugs may also alter the cytotoxicity of the drug. The existing drug metabolism researches mainly focus on the absorption, distribution, and chemical structure changes of drugs *in vivo*, with reduced pharmacological activity, or increased polarity to be more easily excreted after metabolism. It is possible that the drugs are also partially metabolized in the tumor cells to cause properties changes. Therefore, we have developed a method for analyzing intracellular drug metabolism in tumor cells.

Doxorubicin is used in the clinical treatments for breast cancer, leukemia, lymphoma, and sarcomas (Damiani et al., 2016). The mechanism of doxorubicin is known as to intercalate into DNA, leading to DNA breaks (Ross et al., 1978) and interfering DNA replication (Momparler et al., 1976). Previous studies of doxorubicin metabolism were based on the metabolism in liver. Half of doxorubicin is eliminated from the body in the original form. A variety of metabolic enzymes such as Cytochrome P450, aldo/keto reductase superfamily (Bains et al., 2010) and carbonyl reductases (Kassner et al., 2008; Zhou et al., 2015) are involved in the metabolism of doxorubicin. The *in vivo* metabolites of doxorubicin are mainly present in five forms: DOXol, DOX-semiquinone, DOX hydroxyaglycone, DOX deoxyaglycone, and DOXol aglycone (Minotti, 1989; Licata et al., 2000; Mordente et al., 2009). 7-deoxydoxorubicinone and DOXol have been shown to be associated with cardiotoxicity in cardiomyocytes (Minotti et al., 1998; Minotti et al., 2004). Studies on the metabolism of doxorubicin have confirmed that drug metabolites can significantly affect the treatment result of drugs and cause side effects. Besides of studies on cardiac and liver cells, there are few reports of doxorubicin metabolites from other cell lines.

In clinical cancer treatments, prolonged chemotherapies will induce drug-resistant and cause failure of treatments. Significantly reduced cytotoxicity of doxorubicin in drug-resistant cells is thought to be associated with decreased intracellular drug concentration due to overexpression of ABC transporters superfamily such as p-glycoprotein (p-gp) (Stavrovskaya, 2000). In our previous studies, we found that doxorubicin accumulated in the cytosol of doxorubicin-resistant breast cancer cell line MCF7/DOX, whereas in the sensitive cell MCF7/WT doxorubicin is concentrated in the nucleus (Ma et al., 2012). The difference in the distribution of doxorubicin in drug-resistant and sensitive cells suggests unknown protective mechanism may exist in drug-resistant cells. Doxorubicin metabolism affects its excretion and cytotoxicity, and similar metabolic reactions may occur in drug-resistant cells to affect its distribution and activity. Drugs accumulated in tumor cells are difficult to meet the demand for analysis, while drug-resistant tumor cells that can tolerate high drug stress and have more active metabolism are suitable as materials for intracellular drug metabolism study. Therefore, we developed a method to effectively extract doxorubicin intracellular metabolites and figure out unknown metabolites by LC-MS/MS analysis. The structures of unknown metabolites are inferred by multistage mass spectrometry.

## Materials and Methods

### Cell Culture

Human breast cancer cell MCF7 was purchased from ATCC and the multidrug resistance MCF7/DOX cell line was developed based on MCF7 (Yan et al., 2006). Starting from 1/10 of the IC50, the DOX concentration in the medium was gradually increased after the cells were stably grown. After that, MCF7/DOX resistant cell lines with stable drug resistance index of more than 200 times were obtained. Both cell lines were cultured in Dulbecco’s modified Eagle’s medium (DMEM; Invitrogen Corp., CA, United States) supplemented with 10% fetal bovine and 2 U/ml insulin. Cells were grown in incubators at 37°C and 5% CO_2_.

### Preparation of Samples for LC-MS

MCF7/DOX and MCF7/WT cells were cultured in T-75 flasks and exposed to 18 and 100 µg/ml doxorubicin for 12 h. All the cells were observed survival. The cell membrane of the cells was intact and the cells were still alive before disruption. The cells were transferred to a 1.5-ml centrifuge tube and washed three times with PBS.

Riganti, et al. described a condition for doxorubicin extraction, which mixed ethanol and 0.3 N HCl with equal volume (Riganti et al., 2005). In order to extract doxorubicin and its metabolites simultaneously, a variety of extraction conditions based on this method have been investigated, and the 1:1 mixture of alcohol and 0.3 M HCl solution has the highest extraction efficiency. In order to remove the protein from the extraction solution, an equal volume of 40% TCA is required. 30 min, 400 W water bath ultrasound can significantly improve the extraction efficiency compared to vortex and probe-in ultrasound.

Therefore, 250 µl 0.3 M HCl, 250 µl HPLC-grade ethanol, and 500 µl 40% TCA were added to the cell pellet which is harvested from T-75 flasks. The cell suspension was placed in a 400-W cold water bath ultrasonic for 30 min. The supernatant was collected after centrifuged at 12,000 g, 4°C for 30 min, and was keep in 4°C for further LC-MS analysis.

### LC-MS Analysis

Chromatographic separation was performed on ACQUITY UPLC BEH C18 Column (1.7 µm, 2.1 mm × 50 mm) to separate different metabolite components. 0.1% formic acid aqueous solution (phase A)/Methanol (phase B) was selected as mobile phases. The proportions of mobile phase A:B was initially 90:10 and switched to 5% phase A from 0.1 to 9 min, then switch back to 10% of phase B from 11 to 11.5 min and keep 10% phase B until 15 min. The mobile phase was delivered at a rate of 0.2 ml/min. The column temperature was maintained at 20°C. The injection volume is 5 µl (MCF7/DOX) and 2 µl (MCF7/WT). Ultraviolet detection was set at the wavelength 480 nm, which correspond to the specific wavelength of anthracycline structure in Doxorubicin and its metabolites (Cummings et al., 1991). Multi-stage mass spectrometry was performed on LTQ Orbitrap XL in positive ion mode with HESI. Source Voltage was set at 4,300 V, and APCI Vaporizer temperature was 100°C. The capillary voltage was 44 V and the temperature was 300°C. Normalized Collision Energy was 35 eV.

## Results

### Differences in Doxorubicin Metabolism Between Drug-Resistant and Wild-Type MCF7 by LC-MS

**Figure 1:**
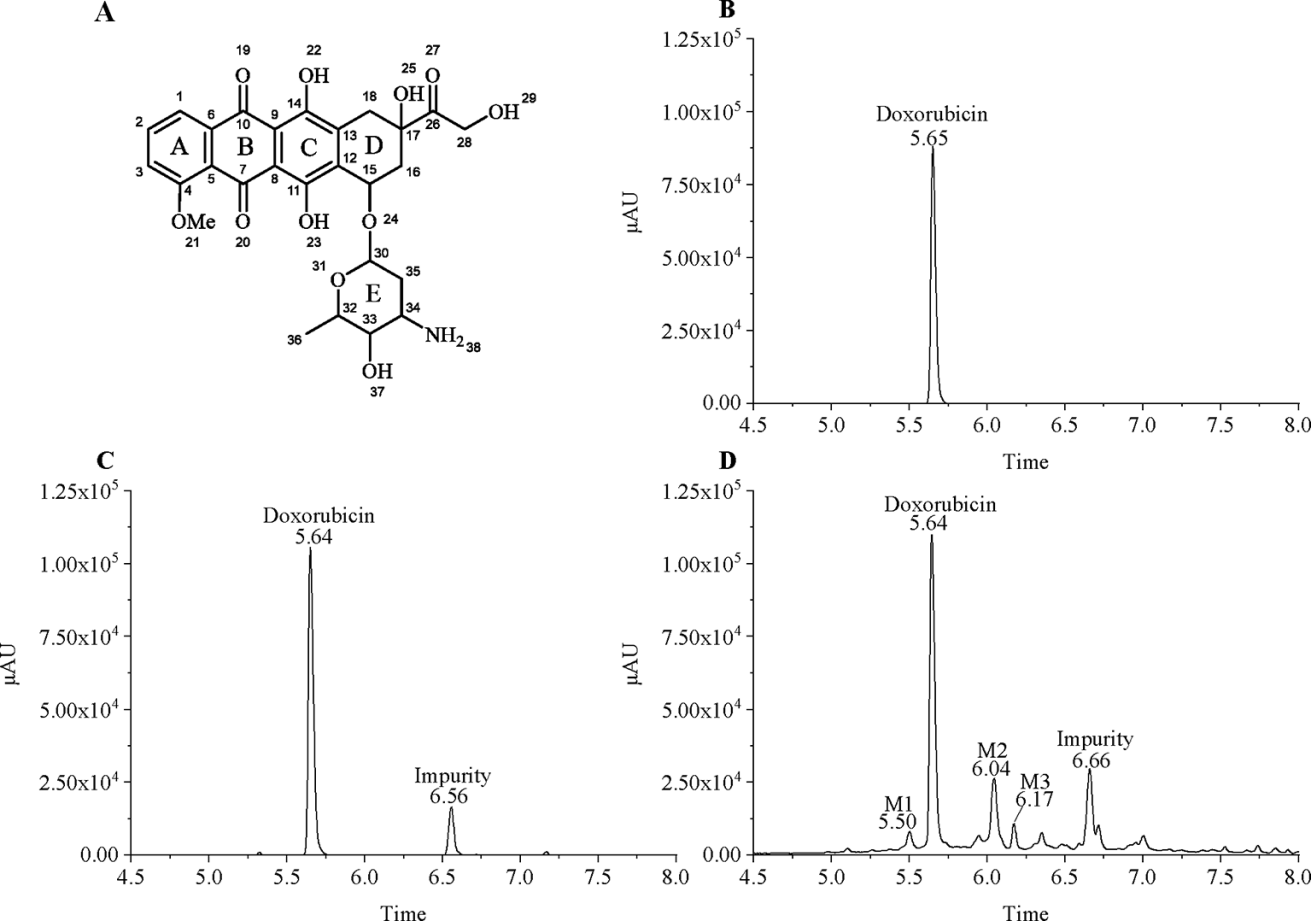
Chromatographic separation of doxorubicin and its intracellular metabolites. **(A)** Chemical structure of doxorubicin; **(B)** 20 µg/ml doxorubicin standard; **(C)** Extract of MCF7/WT treated with 20 µg/ml of doxorubicin; and **(D)** Extract of MCF7/DOX treated with 100 µg/ml of doxorubicin.

Since MCF7 and MCF7/DOX have different sensitivities to doxorubicin, we treated them with 18µg/ml and 100µg/ml doxorubicin, respectively, to ensure that the cells survived during a short period of drug treatment. MCF7 and MCF7/DOX cell lysate extracts were subjected to LC-MS analysis. As the doxorubicin with anthracycline structure (**Figure 1A**) has a unique absorption at 480, we set the UV detector wavelength at 480 nm. Surprisingly, a plurality of peaks having ultraviolet absorption at 480 nm were observed in the drug-resistant cells extracts (**Figure 1**), which indicates that multiple doxorubicin analogues exist in drug-resistant cells. Doxorubicin is eluted at ∼5.64 min and an impurity produced by the contact of doxorubicin with trichloroacetic acid is eluted at ∼6.56 min (MCF7/WT) and ∼6.66 min (MCF7/DOX). Affected by drug efflux, although drug-resistant cells are under greater pressure, the amount of doxorubicin in the drug-resistant cells is less than that of wild-type cells. We increased the injection volume of drug-resistant cell samples by equalizing the peak area of doxorubicin in both cells to compare their metabolic differences. Although the concentration of intracellular doxorubicin is higher, other peaks observed in the chromatogram of drug-resistant cells were not found in wild-type MCF7 cells. Most of these peaks appear after doxorubicin, indicating that the polarity of these metabolites is weaker. Three metabolites (M1–M3) in these metabolites can be better separated by chromatography and have a larger peak area, eluted at 5.50, 6.04, and 6.17 min, respectively. Rest of the metabolites are more difficult to distinguish. The *m/z* of the metabolites M1–M3 in mass spectrum was determined to be 560, 574, and 588, respectively. The molecular weight of M1 differs from doxorubicin (*m/z* = 544) by 16. The molecular weight of M2 is 14 greater than M1, and the molecular weight of M3 is also 14 greater than M2. This suggests that there may be a structural association between the three metabolites.

### Mass Fragmentation Analysis of Doxorubicin Metabolites in MCF-7/DOX

**Figure 2:**
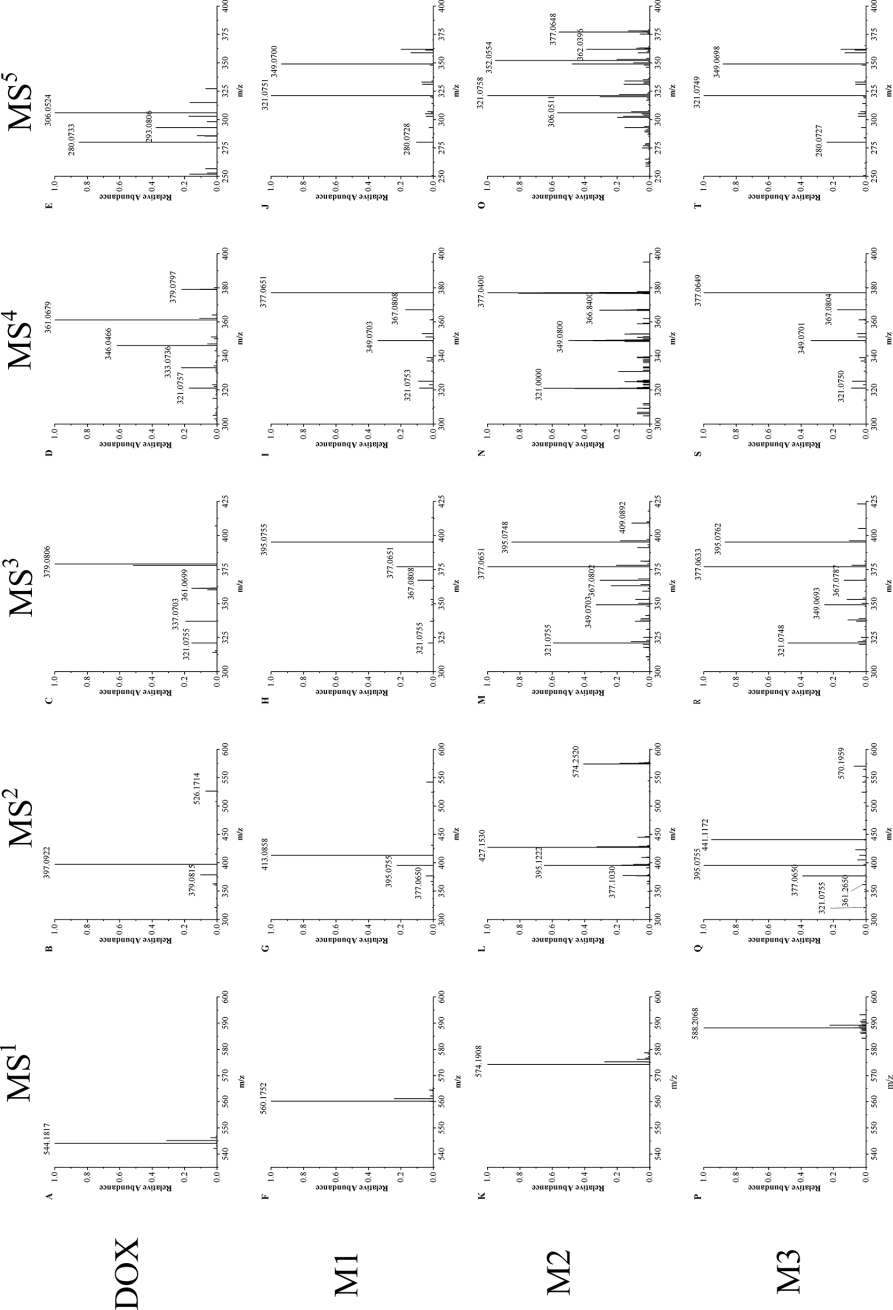
Multistage mass spectra of doxorubicin and its metabolites. **(A**–**E)** MS1∼MS5 mass fragmentation of doxorubicin, **(F**–**J)** MS1∼MS5 mass fragmentation of doxorubicin metabolites M1, **(K**–**O)** MS1∼MS5 mass fragmentation of doxorubicin metabolites M2, **(P**–**T)** MS1∼MS5 mass fragmentation of doxorubicin metabolites M3.

**Figure 2** shows 1 to 5 stages mass spectrum of doxorubicin (DOX) and the selected metabolites (M1–M3) from drug-resistant cells. Ions of doxorubicin (*m/z* = 544) and its metabolites (M1–M3) (*m/z* = 560, 574, and 588) in primary mass spectrometry were selected for further mass fragmentation.

The MS^2^ spectrum of doxorubicin (spectrum B) shows two intense peaks of *m/z* 397 and 379, which corresponds to the loss of the glycoside group (*m/z* = 147) on ring D and a water (*m/z* = 18) in plus. Although the metabolites have different molecular weights, they have a similar cleavage process to doxorubicin, same glycoside group is removed in each metabolite (M1: *m/z* 560 → 413; M2: *m/z* 574 → 427; M3: *m/z* 588 → 441, Δ*m/z* = 147). This result indicate that the glycoside group keeps unchanged in both doxorubicin and its metabolites. Besides this major fragment ion, all the metabolites have two intense peaks of *m/z* 395 and 377, which only have one degree of unsaturation difference from the major fragment ions in doxorubicin at *m/z* 397 and 379. Therefore, in MS^3^, the fragment ions of 413 (M1), 427 (M2) and 441 (M3) were chosen for further fragmentation to verify the source of ion 395 and 377.

MS^3^ result shows that ion 395 and 377 both perform intensely (spectrums H, M, R). They may correspond to a stable and common structure part of doxorubicin metabolites, and they are easily associated with the fragment of doxorubicin 397 and 379. The ions *m/z* of 379 (DOX) and 395 (M1–M3) were then chosen for the MS^4^ fragmentation, respectively. It is found that the fragmentation manner of ion 395 in M1–M3 (spectrum I, N, S) resemble the way of ion 397 (DOX): the loss of water is the dominant cleavage accompanying with series of fragmentation which end at ion 321. This phenomenon confirms that the chosen metabolite ions contain a very reproductive structural part of doxorubicin.

**Figure 3:**
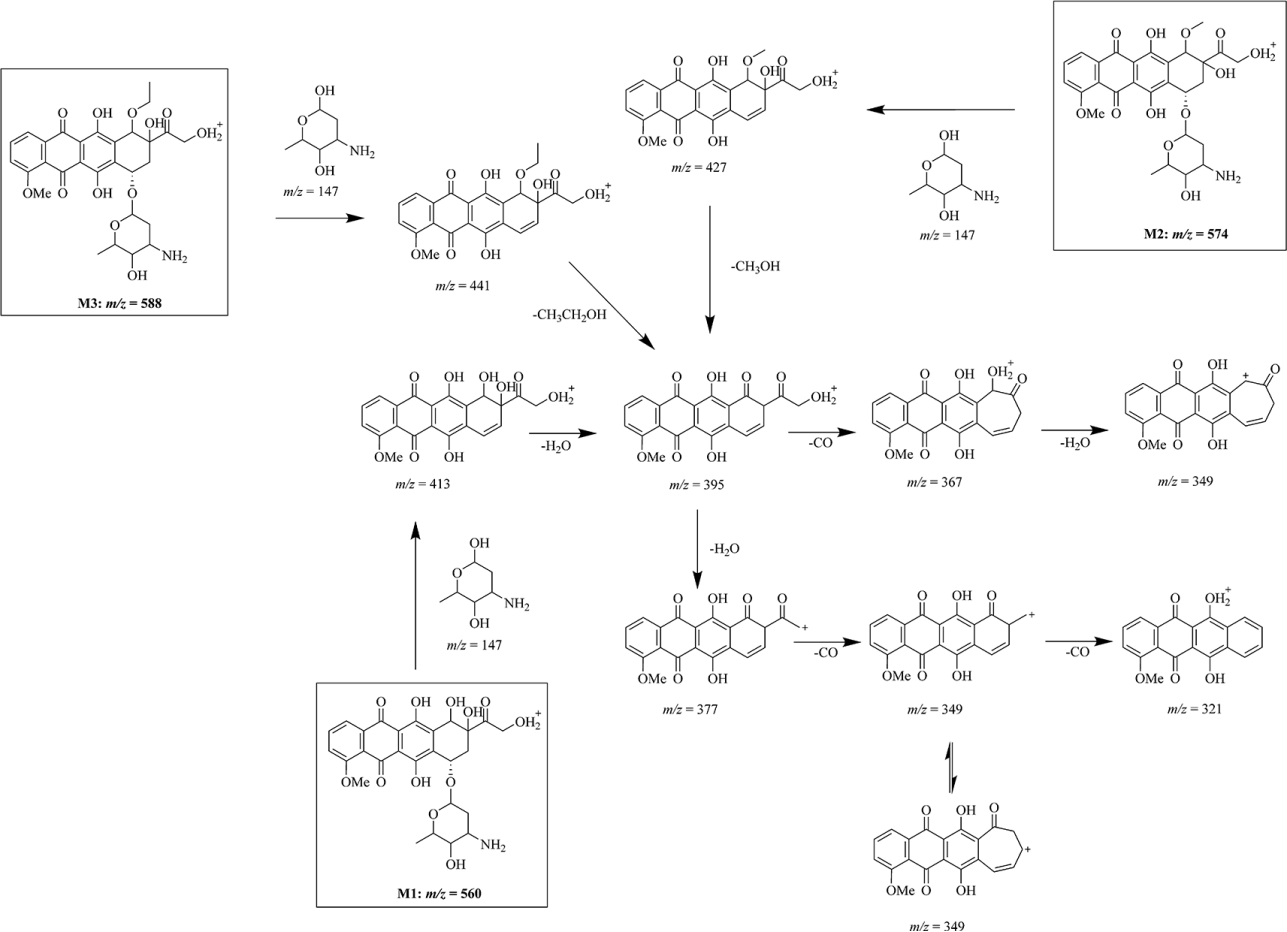
Mass fragmentation pattern of doxorubicin metabolites. Mass fragmentation pattern of doxorubicin metabolites M1 (m/z = 560), M2 (m/z = 574), and M3 (m/z = 588), inferred by multistage mass spectrometry.

In MS^5^, ion of *m/z* 361 (doxorubicin) and 377 (M1–M3) were fragmented, respectively. The ion of *m/z* 321 was observed in both doxorubicin and its metabolites (spectrum E, J, O, T). This confirms that the four-membered ring structure of doxorubicin (**Figure 3**) keeps unchanged in drug and its metabolites. It can also prove that the modification site of the doxorubicin metabolite should be on the ring D or the side chain attached to ring D (**Figure 1A**).

After the multiple stage mass spectrometry analysis, we can infer the fragmentation pathway of doxorubicin as 544 → 397 → 379 → 321. Although the metabolites M1–M3 have different mass, same ion fragments of *m/z* 395, 377, and 321 were observed in multistage mass spectra of different metabolites following identical fragmentation process (560, 574, 588 → 413, 427, 441 → 395 → 377 → 321), demonstrating that the biggest ion fragments of *m/z* 395 are the common structure shared by these metabolites. Compare with the fragmentation manner of ion 397 in doxorubicin, the 395 comes more reasonably from an additional brunch loss on the ring D which induces simultaneously a degree of unsaturation in the system.

### Structure Speculation of Doxorubicin Metabolites

Based on the similarities and differences between the mass fragmentation pattern of doxorubicin and the metabolites, we have inferred the chemical structure of the metabolites and their mass fragmentation pathway in **Figure 3**. After a common loss of glycoside group from the molecular, fragment ions of *m/z* 413, 427, and 441 from three different metabolites M1–M3 give same secondary ion *m/z* 395. According to the high resolution of mass spectrometry analysis, we infer the difference between *m/z* 413, 427, 441 to *m/z* 395 as loss of a water (*m/z* = 18.01), a methanol (*m/z* = 32.03), and an ethanol (*m/z* = 46.04). Profound investigation on the secondary fragment ion of *m/z* 377 in three different metabolites shows that a similar cascade fragmentation of the C = O group occurs on producing series secondary ions of *m/z* 349, 321 by cleavage of CO (*m/z* = 27.99).

As we discussed in the previous part, the glycoside part is not modified in the metabolism. The common fragment ion of *m/z* 321 confirms that either in doxorubicin or its metabolites, the major part of anthracycline keeps unchanged, especially on ring A, B, and C. The possible and unique modifiable part in drug should be its activity part on ring D. All the evidence indicates that three metabolites we found in drug resistant cells may be the products of one same metabolism pathway: an oxidation of drug (M1) followed by series of methylation (M2, M3).

The methylation is ordinary for a hydroxyl-rich molecular such as doxorubicin. Therefore, the location of the oxidation is the key point for the structural expose of these metabolites. It is not a stable compound structure if the modification occurs at position 28, so the modification site of M1 can only be on the D ring. To guarantee the cleavage of glycosidic group, position 15 and 16 cannot be modified, which mean the only possible modification site is at position 18. We also have demonstrated that the fragment *m/z* 395 is identical in mass spectra of different metabolites. It is worth noting that *m/z* 395 only has one degree of unsaturation differ to the *m/z* 397 in doxorubicin. We have reason to believe that the modification on ring D induces this slight difference on the fragment ions when it cleaves. Therefore, the intracellular metabolism may import a hydroxyl group, a methoxy group and an ethoxy group at the 18 position of doxorubicin, respectively, to produce the M1, M2, and M3 (**Figure 3**).

## Discussion

Doxorubicin is an anthracycline type antitumor agent isolated from *Streptomyces paucities* (Malla et al., 2010). It is now widely used in clinical chemotherapy for a variety of tumors. However, the side effects of doxorubicin seriously affect its clinical application, resulting in chemotherapy failure (drug resistance) (Smith et al., 2006) and cardiotoxicity (Chen et al., 2011). Cardiotoxicity of doxorubicin is associated with a variety of doxorubicin *in vivo* metabolites, and doxorubicin resistance has been shown to be associated with drug efflux of p-gp. Most studies on doxorubicin now focus on killing tumor cells, but the doxorubicin in tumor cells may also be metabolized into functionally different metabolites like cardiomyocytes. The study of intracellular drug metabolism is helpful in complementing the mechanism of pharmacodynamic effect. However, we did not see much reports of intracellular drug metabolism in tumors.

The MCF7 doxorubicin-resistant cells we used were obtained from doxorubicin-induced MCF7 wild-type cells, and it is also resistant to many other drugs, such as mitoxantrone and epirubicin. Therefore, MCF7/DOX cells are a multidrug resistant cell (Yan et al., 2006). In previous studies, we found that doxorubicin is localized differently in MCF7 and MCF7/DOX: a certain amount of doxorubicin is still localized in the cytosol in the MCF7/DOX, while in the sensitive cells the drugs are concentrated in the nucleus. We determined doxorubicin localization by observing the fluorescence of the anthracycline structure of doxorubicin (Ma et al., 2012). Vesicles play an important role in this localization change, and we also speculate that doxorubicin may have modifications that do not affect its anthracycline structure but affect its physical and chemical properties.

We have developed an intracellular trace metabolites extraction and LC-MS analysis method to identify doxorubicin metabolites in drug-resistant and sensitive cells. After high-resolution multistage mass spectrometry was used to detect doxorubicin structural analogs, we found that the fragmentation processes of these metabolites have commonalities and the structures inferred by mass fragmentation pattern are similar. We suspect that doxorubicin is modified in the drug-resistant cell by a series of related metabolic enzymes. Although only three of these metabolites are mentioned, there are still many suspected metabolites with m/z 395 → 377 characteristic mass fragmentation pattern that have not yet been analyzed. After drug treatment for different time (4–36 h), intracellular doxorubicin and its metabolites gradually accumulate over time, with no other differences.

Our work is limited by the sensitivity of the analytical method, low content of metabolites, and the complex composition. In addition, residual impurities also affect chromatographic separation and sensitivity of mass spectrometry detection. It’s difficult to separate and purify the metabolites. Therefore, the toxicity and polarity of the currently obtained intracellular metabolites of doxorubicin are still unknown. In future work, we will continue to improve the method to separate and identify metabolites. Finding enzymes involved in this metabolic process is also very important in the future study. Based on current results, hydroxylase and hydroxymethylase are involved in the metabolic processes, but it is not yet certain which specific enzymes are included. The currently known metabolism of doxorubicin is mainly mediated by the cytochrome P450. in addition, carbonyl reductase and aldo-keto reductase have also been shown to be involved in some metabolic processes. But none of these enzymes catalyze metabolic reactions to get these newly discovered metabolites. In future studies, it is difficult but important to isolate and identify intracellular metabolites of tumor cells and infer their chemical reaction processes to determine the enzymes involved in this metabolism. The identification of these metabolic enzymes can complement the mechanism of drug resistance. In this study, although MCF7/DOX is a multidrug-resistant cell, we have only tested drug-resistant cells treated with doxorubicin because the specific structure of doxorubicin is easy to observe. In the future, we will also use the method developed based on doxorubicin to detect the intracellular metabolites of tumor cells of other antitumor drugs. We hope to find commonalities in different drug metabolism in tumor cell metabolism, and to confirm whether this metabolic process is universal.

## Conclusion

The discovery of new drug metabolites in tumor cells suggests that drugs in the cells may not all present as original form. Our results indicate that doxorubicin has been modified in drug-resistant tumor cells, and these metabolites are different from metabolites *in vivo*, suggesting that drug-resistant cells may have unique drug metabolism mechanisms.

## Data Availability Statement

The datasets analyzed in this manuscript are not publicly available. Requests to access the datasets should be directed to wangxujn@foxmail.com.

## Author Contributions

XW, RH, and JJ conceived and designed the research. XW, WW, and YujC preformed the experiments. XW wrote the manuscript, and XG and RH have helped in revising the manuscript. JJ and YunC have given administrative, technical, and material support.

## Funding

This study was supported by the National Natural Science Foundation of China (Grant Nos. 30772586 and 81361168001) and The Clinical Science and Technology Projects (Grant No. BL2014019).

## Conflict of Interest

The authors declare that the research was conducted in the absence of any commercial or financial relationships that could be construed as a potential conflict of interest.

## Abbreviations

DOX, Doxorubicin; LC-MS, liquid chromatography-mass spectrograph; p-gp, p-glycoprotein.
