# Corrigendum: Discovery of Novel Doxorubicin Metabolites in MCF7 Doxorubicin-Resistant Cells

**DOI:** 10.3389/fphar.2020.603491

**Published:** 2020-11-26

**Authors:** Xu Wang, Renjie Hui, Yun Chen, Wentao Wang, Yujiao Chen, Xiaohai Gong, Jian Jin

**Affiliations:** School of Pharmaceutical Sciences, Jiangnan University, Wuxi, China

**Keywords:** drug resistant, doxorubicin, intracellular drug metabolism, LC-MS/MS, breast cancer

In the original article, there were several errors. A correction has been made to:

Section **Materials and Methods**, sub-section **Cell Culture**, paragraph 1:

“Human breast cancer cell MCF7 was purchased from ATCC and the multidrug resistance MCF7/DOX cell line was developed based on MCF7 (Yan et al., 2006). Starting from 1/10 of the IC50, the DOX concentration in the medium was gradually increased after the cells were stably grown. After that, MCF7/DOX resistant cell lines with stable drug resistance index of more than 200 times were obtained. Both cell lines were cultured in Dulbecco’s modified Eagle’s medium (DMEM; Invitrogen Corp., CA, United States) supplemented with 10% fetal bovine and 2 U/ml insulin. Cells were grown in incubators at 37°C and 5% CO_2_.”

Section **Materials and Methods**, sub-section **LC-MS Analysis**, paragraph 1:

“Chromatographic separation was performed on ACQUITY UPLC BEH C18 Column (1.7 µm, 2.1 mm × 50 mm) to separate different metabolite components. 0.1% formic acid aqueous solution (phase A)/ Methanol (phase B) was selected as mobile phases. The proportions of mobile phase A:B was initially 90:10 and switched to 5% phase A from 0.1 to 9 min, then switch back to 10% of phase B from 11 to 11.5 min and keep 10% phase B until 15 min. The mobile phase was delivered at a rate of 0.2 ml/min. The column temperature was maintained at 20°C. The injection volume is 5 μl (MCF7/DOX) and 2 μl (MCF7/WT). Ultraviolet detection was set at the wavelength 480 nm, which correspond to the specific wavelength of anthracycline structure in Doxorubicin and its metabolites (Cummings et al., 1991). Multi-stage mass spectrometry was performed on LTQ Orbitrap XL in positive ion mode with HESI. Source Voltage was set at 4,300 V, and APCI Vaporizer temperature was 100°C. The capillary voltage was 44 V and the temperature was 300°C. Normalized Collision Energy was 35 eV.”

Section **Discussion**, paragraph 3:

“We have developed an intracellular trace metabolites extraction and LC-MS analysis method to identify doxorubicin metabolites in drug-resistant and sensitive cells. After high-resolution multi-stage mass spectrometry was used to detect doxorubicin structural analogs, we found that the fragmentation processes of these metabolites have commonalities and the structures inferred by mass fragmentation pattern are similar. We suspect that doxorubicin is modified in the drug-resistant cell by a series of related metabolic enzymes. Although only three of these metabolites are mentioned, there are still many suspected metabolites with m/z 395 → 377 characteristic mass fragmentation pattern that have not yet been analyzed. After drug treatment for different time (4–36 h), intracellular doxorubicin and its metabolites gradually accumulate over time, with no other differences.”

The authors apologize for these errors and state that this does not change the scientific conclusions of the article in any way. The original article has been updated.

